# Roles of Neuropeptides, VIP and AVP, in the Mammalian Central Circadian Clock

**DOI:** 10.3389/fnins.2021.650154

**Published:** 2021-04-15

**Authors:** Daisuke Ono, Ken-ichi Honma, Sato Honma

**Affiliations:** ^1^Department of Neuroscience II, Research Institute of Environmental Medicine, Nagoya University, Nagoya, Japan; ^2^Department of Neural Regulation, Nagoya University Graduate School of Medicine, Nagoya, Japan; ^3^Research and Education Center for Brain Science, Hokkaido University Graduate School of Medicine, Sapporo, Japan

**Keywords:** circadian rhythm, suprachiasmatic nucleus, AVP, VIP, neuronal coupling, synchronization, entrainment

## Abstract

In mammals, the central circadian clock is located in the suprachiasmatic nucleus (SCN) of the hypothalamus. Individual SCN cells exhibit intrinsic oscillations, and their circadian period and robustness are different cell by cell in the absence of cellular coupling, indicating that cellular coupling is important for coherent circadian rhythms in the SCN. Several neuropeptides such as arginine vasopressin (AVP) and vasoactive intestinal polypeptide (VIP) are expressed in the SCN, where these neuropeptides function as synchronizers and are important for entrainment to environmental light and for determining the circadian period. These neuropeptides are also related to developmental changes of the circadian system of the SCN. Transcription factors are required for the formation of neuropeptide-related neuronal networks. Although VIP is critical for synchrony of circadian rhythms in the neonatal SCN, it is not required for synchrony in the embryonic SCN. During postnatal development, the clock genes *cryptochrome (Cry)1* and *Cry2* are involved in the maturation of cellular networks, and AVP is involved in SCN networks. This mini-review focuses on the functional roles of neuropeptides in the SCN based on recent findings in the literature.

## Introduction

Creatures on earth anticipate cyclic changes in the surrounding environment, such as day–night, seasonal, and annual changes, to adapt their own physiological functions so that they can minimize risks to survival. Among them, rhythmic changes adapting to environmental changes caused by the rotation of the earth are driven by intrinsic oscillatory mechanisms called the circadian clock. In mammals, the circadian system comprises a hierarchical structure involving the master clock in the brain and a number of peripheral clocks situated throughout the body. Almost all cells in our body possess a circadian clock in which the molecular machinery, namely, transcription–translation feedback loops involving several clock genes and their protein products, generates circadian rhythmicity (Reppert and Weaver, 2002). Circadian rhythms of cells and tissues are coordinated by the master circadian clock located in the suprachiasmatic nucleus (SCN) of the hypothalamus (Weaver, 1998). The SCN is the only clock that can entrain to the environmental light–dark (LD) time cue from the retina *via* the retinohypothalamic tract (RHT) (LeGates et al., 2014). Ultimately, the circadian clock in the SCN regulates rhythms within a variety of physiological output functions such as sleep/wakefulness, body temperature, and endocrine functions.

The SCN contains approximately 20,000 neurons that show circadian rhythms in clock gene expression, cytosolic Ca^2+^, and spontaneous firing, and the rhythms are also maintained in culture conditions, in the slice and in dispersed cells (Welsh et al., 1995; Ikeda et al., 2003; Yamaguchi et al., 2003). The distribution of the circadian period from dispersed SCN cells is larger than that from slice cultures (Herzog et al., 2004; Honma et al., 2004). Furthermore, the robustness of circadian rhythmicity in dispersed cells is weaker than that in slices (Liu et al., 2007; Ono et al., 2013). These results suggest that cellular networks are crucial for coherent circadian rhythms in the SCN.

## Heterogeneous Cell Types in the Suprachiasmatic Nucleus

**FIGURE 1:**
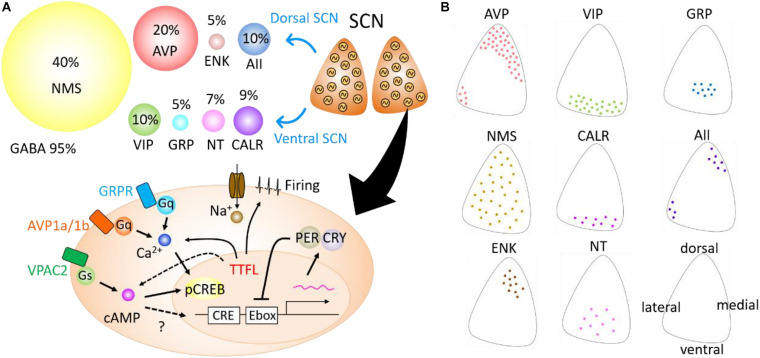
Neuropeptides expressed in the suprachiasmatic nucleus (SCN) and intracellular signaling. **(A)** A variety of neuropeptides expressed in the SCN (top). Cells with an autonomous circadian oscillator are schematically shown in the SCN in coronal plane. Circles of different sizes indicate the percentage of neurons expressed in the SCN. Arginine vasopressin (AVP), enkephalin (ENK), and angiotensin II (AII) are expressed mainly in the dorsal area of the SCN, whereas vasoactive intestinal polypeptide (VIP), gastrin-releasing peptide (GRP), neurotensin (NT), and calretinin (CALR) are mainly expressed in the ventral SCN. Neuromedin-S (NMS) is broadly expressed in the SCN, and gamma aminobutyric acid (GABA) in almost all SCN neurons. Schematic view of the intracellular pathways mediating neuropeptide signals in the SCN (bottom). Neuropeptides bind G-protein-coupled receptors (Gq, Gs) and modulate second messenger signaling such as cAMP or Ca^2+^. These signals facilitate the phosphorylation of CREB proteins and change the transcription of *Per* genes. Some signaling from transcription–translation feedback loop (TTFL) could modulate cAMP or Ca^2+^ rhythms. **(B)** Schematic drawing demonstrating the distribution of eight types of peptidergic neurons within the unilateral coronal SCN slice. Directions are shown in the lower right SCN.

The SCN is, neuroanatomically and cytochemically, divided into two subregions—dorsal and ventral subregions (Figure 1). The ventral SCN receives major afferent pathways, including those from the retina, midbrain raphe nucleus, and intergeniculate leaflet (Hattar et al., 2006; McNeill et al., 2011). It contains vasoactive intestinal peptide (VIP), gastrin-releasing peptide (GRP), neurotensin (NT), and calretinin (CALR)-expressing neurons (Abrahamson and Moore, 2001). Arginine vasopressin (AVP), met-enkephalin (mENK), and angiotensin II (AII)-expressing neurons are located in the dorsal SCN.

Taking advantage of bioluminescence reporters, circadian rhythms can be recorded in individual SCN cells. In dispersed cell culture, individual SCN cells show autonomous circadian rhythms, but not all cells maintain rhythmicity. Webb et al. (2009) measured PER2:LUC bioluminescence from dispersed SCN cells and performed *post hoc* immunostaining to identify which neurons (i.e., those expressing AVP or VIP) are responsible for rhythm generation. They found that the circadian period and percentage of rhythmic cells did not differ between AVP and VIP neurons and concluded that intrinsic circadian oscillation was not restricted to a single class of neuropeptidergic neurons in the SCN. They also treated SCN slices with tetrodotoxin (TTX) for 6 days twice at 6-day intervals and examined PER2:LUC rhythmicity. Since TTX suppresses neural communication, neurons exhibiting circadian rhythms *via* neural inputs no longer exhibit circadian rhythms following TTX treatment, while neurons with a cell-autonomous circadian clock continue to show circadian PER2:rhythms even under TTX treatment. If circadian peacemaking neurons are unique to certain cell types, repeated TTX treatments would reveal circadian rhythms in the same cells. However, the results showed that the cells exhibiting circadian rhythms under TTX treatment were not the same between the two trials. Furthermore, the location of neurons that maintained circadian rhythms in both TTX treatments was not regionally specific; rather, these neurons were scattered throughout the SCN. These results suggest that neurons throughout the SCN are intrinsic but unstable circadian oscillators that are capable of showing robust oscillation by neural networks. A recent study by Hirata et al. (2019) demonstrated that by culturing dispersed SCN cells on microfabricated islands, a substantial number of solitary SCN neurons with no contact with other cells exhibited robust circadian rhythms with regard to clock gene expression and intracellular Ca^2+^ levels. Since TTX treatment suppresses action potential, neuronal firing may itself affect the circadian molecular rhythms in solitary SCN neurons.

## Roles of Vasoactive Intestinal Polypeptide Neurons in the Suprachiasmatic Nucleus

VIP is known as a synchronizer of neuronal networks in the SCN, and approximately 10% of the SCN consists of VIP-positive neurons (Abrahamson and Moore, 2001). Mice lacking VIP or VIP receptor 2 (VPAC2) show desynchronized circadian rhythms in SCN neurons in culture as well as attenuated behavioral rhythms (Harmar et al., 2002; Colwell et al., 2003; Aton et al., 2005; Maywood et al., 2006). Similarly, the application of an excessive amount of VIP onto cultured SCN slices also attenuates the synchrony of cellular circadian rhythms (An et al., 2013). The rhythmic release of VIP in cultured SCN slices has been previously reported (Shinohara et al., 1995; Shinohara et al., 1999). These results suggest that rhythmic release of VIP is important for synchrony of circadian rhythms in SCN neurons. Chemogenetic activation of VIP neurons also changes the spatiotemporal patterns of PER2:LUC rhythms, with lengthening of the circadian period and attenuated amplitude of circadian rhythms (Brancaccio et al., 2013). Maywood et al. (2011) demonstrated that paracrine signaling in the SCN is critical for the synchronization of circadian rhythms. The authors cultured VIP or VPAC2 knockout (KO) SCN containing a PER2:LUC reporter with a wild-type (WT) graft SCN, and they placed a semipermeable membrane that only passed small molecules, such as neuropeptides, between two SCN slices. Importantly, the circadian rhythms of VIP KO SCN were restored by coculture of WT SCN, indicating that small diffusible molecules such as neuropeptides from the WT SCN are critical for rhythm synchronization. However, it took longer to restore the circadian rhythms of VPAC2 KO SCN as compared to VIP KO SCN. These results indicate that VIP and other molecules, such as AVP and GRP, are crucial for the synchronization of circadian rhythms in the SCN.

VIP and gamma aminobutyric acid (GABA) signaling work synergistically to sustain circadian phase differences in the SCN. For example, a long-day photoperiod shortens the activity time and changes cellular coupling in the SCN (Inagaki et al., 2007). Interestingly, under a long-day photoperiod, the circadian phase of PER2:LUC rhythms between the dorsal and ventral SCN is decoupled at the beginning of culture, which gradually resynchronizes under culture conditions (Evans et al., 2013; Myung et al., 2015). This resynchronization is blocked by a VIP or GABA_*A*_ receptor antagonist. VIP receptor antagonists attenuate both the advance and delay portions of the coupling response curve (i.e., the resetting responses of SCN core neurons as compared to the initial phase relationship with SCN shell neurons). However, in this study, the GABA_*A*_ receptor antagonist mainly attenuated the advance portion of the curve. Furthermore, the effects of GABA receptor antagonists are not evident for circadian rhythms in the cultured WT SCN (Aton et al., 2006; Ono et al., 2019), though GABA dramatically restores the synchrony of circadian rhythms in the VIP KO cultured SCN (Freeman et al., 2013).

Application of VIP into the culture medium induces a phase-dependent phase delay shift in the circadian PER2:LUC rhythm of the SCN slice (An et al., 2011). The VIP-mediated phase shift could be due to cAMP signaling because VIP application increases intracellular cAMP and activity of the cAMP response element (CRE) using cAMP FRET sensor and CRE-*luc* reporter, respectively (An et al., 2011; Hamnett et al., 2019). In addition, extracellular signal-regulated kinases (ERKs) 1/2 and dual specificity phosphatase (DUSP) 4 signaling may also be involved in the VIP-induced responses of circadian rhythms in the SCN, which is independent of the cAMP response element-binding protein (CREB) pathway (Hamnett et al., 2019). Similarly, optogenetic activation of VIP neurons causes a phase delay shift in circadian PER2:LUC rhythms in the SCN slice as well as behavioral rhythms (Mazuski et al., 2018; Patton et al., 2020). Intriguingly, Mazuski et al. (2018) found that VIP neurons showed tonic or irregular firing patterns and optogenetically mimicked irregular firing induced large phase shifts in both PER2:LUC rhythms in the SCN and behavioral rhythms. This result shows the physiological significance of irregular firing in SCN VIP neurons for the entrainment of circadian rhythms.

VIP neurons exhibit circadian rhythms during spontaneous firing in cultured SCN slices (Hermanstyne et al., 2016). Taking advantage of the *in vivo* fiber photometry method, Jones et al. (2018) demonstrated that VIP neurons exhibit circadian rhythms in spontaneous calcium activity *in vivo* under LD and constant dark (DD) conditions, but not under constant light (LL) conditions. In addition, calcium activity was evoked by light pulses with daily peaks occurring at approximately CT 12, which is consistent with light-induced circadian phase shifts. Chemogenetic suppression of neuronal activity of VIP neurons attenuates light-induced phase shifts. These results indicate that VIP neurons are important for light-mediated resetting of circadian rhythms.

VIP neurons may play an important role in the circadian output rhythms. Optogenetic activation of VIP neurons in the SCN suppresses paraventricular nucleus (PVN) neurons *via* GABA release, and chemogenetic suppression of these neurons in turn increases corticosterone concentration (Paul et al., 2020). This regulation could be mediated by corticotropin-releasing factor (CRF) neurons in the PVN because activation of SCN neurons suppresses CRF neuronal activity (Ono et al., 2020). The SCN to PVN neuronal pathways also regulate sleep and wakefulness (Ono et al., 2020). VIP neurons regulate nighttime sleep called “siesta” in mice, which is due to a specific group of VIP neurons that is active during nighttime in the SCN (Collins et al., 2020). Recently, single-cell RNA sequencing analysis revealed two types of VIP neurons in the SCN: pacemaker and non-pacemaker cells. Such techniques help categorize cell types in the SCN that regulate a variety of physiological functions (Todd et al., 2020; Wen et al., 2020).

## Roles of Arginine Vasopressin Neurons in the Suprachiasmatic Nucleus

Approximately 20% of SCN consists of AVP-positive neurons, which is the second largest peptide population in the SCN (Abrahamson and Moore, 2001). The *Avp* promoter contains an E-box element, which is the target of CLOCK/BMAL1 transcription factors, and *Avp* mRNA levels exhibit a robust daily rhythm with daytime peak (Uhl and Reppert, 1986; Jin et al., 1999). *Avp-Eluc* reporter mice also show circadian *Avp* expression rhythms in SCN slice (Yoshikawa et al., 2015). Since the *Avp* promoter contains an E-box, *Avp* mRNA levels in the SCN are low throughout the day in *Clock* mutant mice (Jin et al., 1999). CRY1 and CRY2 are negative elements of E-box-related transcription. Thus, *Avp* expression is expected to increase without CRYs. However, *Avp* expression was suppressed and arrhythmic in the SCN of *Cry1* and *Cry2* double-deficient (*Cry1/Cry2* KO) mice, suggesting that suppression of E-box-related transcription is not only due to *Cry* genes.

Although VIP is a strong synchronizer in the SCN, the AVP functions as a weak synchronizer. For example, the circadian rhythms of VPAC2 KO SCN are restored by the coculture of WT SCN, but this restoration is inhibited by the application of AVP receptor antagonists (Maywood et al., 2011). Edwards et al. (2016) demonstrated that the arrhythmicity of *Cry1/Cry2* KO SCN was restored by AAV-mediated transduction of *Cry1*. Interestingly, treatment of *Cry1/Cry2* KO SCN with AVP receptor antagonists only induced low-amplitude circadian rhythms when applied simultaneously with *Cry1* gene transduction, but it did not attenuate restored rhythms when treated after circadian rhythms were restored by *Cry1* transduction, suggesting that AVP signaling is required for the induction, but not maintenance, of CRY-dependent circadian rhythms in *Cry1/Cry2* KO SCN (Edwards et al., 2016). Loss of the AVP receptors V1a and V1b in the SCN can modify neuronal networks in the SCN. The clock gene, *Per1*, expression rhythm in the SCN is known to show a phase gradient with phase-leading at the dorsomedial SCN and -lagging at the ventrolateral area (Yamaguchi et al., 2003). This spatiotemporal pattern was perturbed by the application of cycloheximide (CHX), a translation inhibitor, and restored after washout in WT mice, but not in V1a and V1b KO mice (Yamaguchi et al., 2013). These mice show immediate re-entrainment to a new LD environment after an abrupt shift in LD cycles, suggesting that V1a and V1b confer SCN resistance to external perturbation.

The functional roles of the circadian clock of AVP neurons in the SCN were evaluated by genetically manipulating AVP neurons using the Cre-loxP system. AVP neuron-specific *Bmal1* KO (*Avp-Bmal1^–/–^*) mice showed longer activity time with lengthening of the free-running period than WT mice (Mieda et al., 2015), but VIP neuron-specific *Bmal1* KO did not affect circadian behavioral rhythms (Lee et al., 2015). PER2:LUC rhythms in the dorsal SCN of *Avp-Bmal1^–/–^* mice exhibited a lengthened circadian period and attenuated amplitude (Mieda et al., 2015) and failed to resynchronize circadian rhythms in the SCN after the washing out of TTX (Shan et al., 2020). Lee et al. (2015) demonstrated similar results in *neuromedin-S* (NMS) Cre mice. Overexpression of PER2 or deletion of *Bmal1* in NMS neurons in the SCN abolished circadian wheel-running activity rhythms under constant darkness (DD). NMS KO, *per se*, did not change circadian behavioral rhythms, indicating that NMS itself has no role in the SCN circadian clock. Approximately 40% of the SCN consists of NMS-positive neurons, and VIP and AVP are coexpressed in NMS neurons. These results suggest that AVP neurons may modulate the coupling of the SCN network for morning and evening behavioral rhythms.

AVP neurons are also important for anticipatory thirst prior to sleep (Gizowski et al., 2016). Optogenetic activation of AVP neurons in the SCN increased water intake around ZT23 *via* AVP release in the organum vasculosum laminae terminalis (OVLT), an important brain area for water intake. In contrast, optogenetic suppression of neuronal activity in AVP neurons decreased water intake. These results suggest that the anticipatory thirst prior to sleep is driven by excitatory peptidergic neurotransmission mediated by AVP release in the SCN.

## Neuropeptide-Related Neuronal Networks in the Suprachiasmatic Nucleus During Development

During development, dramatic changes in genetic, cellular, and circuit levels were observed in the SCN. Neurogenesis mainly occurs in the SCN around embryonic days 11–15 in mice (Shimada and Nakamura, 1973; Okamura et al., 1983; Kabrita and Davis, 2008; Shimogori et al., 2010), and synaptogenesis occurs rapidly around postnatal days 4–10 (Moore and Bernstein, 1989). Transcription factors such as *Six3*, *Six6*, *Lhx1*, and *Fzd5* are expressed during the embryonic period in the SCN (Shimogori et al., 2010; VanDunk et al., 2011). AVP and VIP mRNA are observed from around embryonic days 17–18 (Romero and Silver, 1990; Isobe and Muramatsu, 1995; Ban et al., 1997; VanDunk et al., 2011). Interestingly, *Six6*-deficient mice show weak wheel-running rhythms and no clear AVP and VIP expression in the SCN (Clark et al., 2013). *Six6-Cre*-dependent *Lhx1* deficiency (*Six3-Cre*;*Lhx1^*loxp*/lox*p*^*) attenuates neuropeptide expression in the SCN, including *Avp*, *Vip*, *Grp*, *Prok2*, *Enk*, and *Nms* (Bedont et al., 2014). In a study of *Six3-Cre*;*Lhx1^*loxp*/lox*p*^* mice, synchrony of circadian PER2:LUC rhythms in the SCN was suppressed and the circadian rhythmicity of wheel-running rhythms was attenuated; these results are phenotypically similar to *Vip* or VPAC2-deficient mice (Harmar et al., 2002; Colwell et al., 2003). *Ror*α*-Cre*;*Lhx1l*^*oxP/loxP*^ mice also exhibited similar circadian phenotypes (Hatori et al., 2014). These results suggest that transcription factors are required for the maturation of neuronal networks in the SCN.

Circadian rhythms in the SCN have been observed before birth. For example, day/night differences in 2-deoxyglucose uptake were observed at embryonic day 19 *in vivo* (Reppert and Schwartz, 1983). *Per1* expression or PER2:LUC rhythms in the SCN are detectable around embryonic days 15–18 (Shimomura et al., 2001; Ansari et al., 2009; Wreschnig et al., 2014; Landgraf et al., 2015; Carmona-Alcocer et al., 2018). Notably, in the fetal SCN, synchronization of circadian PER2:LUC rhythms was not affected by the sodium channel inhibitor, TTX, VPAC2 receptor antagonist, GABA_*A*_ receptor antagonist, or gap junction inhibitor, suggesting that VIP, GABA, gap junction, and neuronal firing are not required for synchronization of circadian rhythms in the fetal SCN (Carmona-Alcocer et al., 2018).

VIP neurons are suggested to be important for the maturation of neuronal networks in the SCN during development. Ablation of VIP neurons in the adult SCN using AAV-mediated expression of caspase3 induced shortening of circadian behavioral rhythms (Mazuski et al., 2020), but only 15% of mice became behaviorally arrhythmic. In contrast, 50% of the mice were arrhythmic when VIP or VPAC2 was deleted globally. In addition, ablation of VIP neurons in the neonatal SCN significantly dampens the circadian PER2:LUC rhythm (Mazuski et al., 2020). These results suggest that VIP neurons are important for synchrony in the SCN during early development but not in adulthood.

On the other hand, Todd et al. (2020) reported that even in adult mice, ablation of VIP neurons in the SCN by AAV-mediated diphtheria toxin A subunit (DTA) expression profoundly disrupted circadian locomotor activity recorded by a telemetry transmitter and running wheel. The difference in behavioral phenotypes in the two studies might be related to the number of VIP neurons spared from deletion and recording methods. The role of VIP neurons in circadian behavioral rhythms is still controversial and remains to be studied.

**FIGURE 2:**
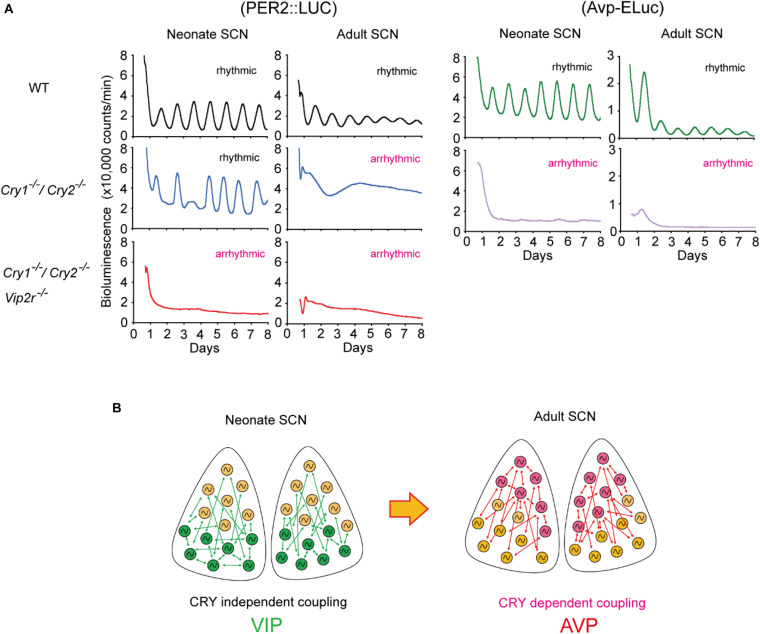
Developmental changes in cellular coupling mechanisms in the suprachiasmatic nucleus (SCN). **(A)** Tissue-level PER2:LUC bioluminescence from the cultured SCN in neonate or adult mice (left). Wild-type (WT) SCNs show circadian rhythms in both neonatal and adult SCNs. *Cryptochrome (Cry)1^– /–^/Cry2^– /–^* SCNs show robust circadian rhythms in the neonate SCN, but not in the adult SCN. Whereas *Cry1^– /–^/Cry2^– /–^/vasoactive intestinal polypeptide (Vip)2r^– /–^* SCN is arrhythmic both in the neonate and adult, which is due to desynchronization of cellular circadian rhythms. *Arginine vasopressin (Avp)-ELuc* bioluminescence from the cultured SCN in neonate or adult mice (right). Circadian *Avp* expression rhythms are observed in the WT SCN, but they are arrhythmic and expression is attenuated in *Cry1^– /–^/Cry2^– /–^* SCN both at neonatal and adult periods. **(B)** Schematic view of a model of cellular coupling in the SCN during postnatal development. CRY1 and CRY2 are involved in the developmental shift from CRY-independent (*via* VIP) to CRY-dependent (*via* AVP) networks in the SCN. Green and pink circles indicate VIP and AVP neurons, respectively. Green and pink arrows indicate signaling *via* VIP and AVP, respectively. This shift is observed around postnatal days 14–21. Modified from Ono et al., 2016.

The importance of neuropeptides and clock gene, *Cry*, for coherent circadian rhythms in the SCN during postnatal development has also been reported. *Cry1* and *Cry2* are thought to be essential genes for circadian rhythms (Okamura et al., 1999; van der Horst et al., 1999; Albus et al., 2002; Yamaguchi et al., 2003). However, circadian *Per1-luc*, PER2:LUC, and spontaneous firing rhythms have been observed in single SCN cells in *Cry1/Cry2*-deficient mice (Maywood et al., 2011; Ono et al., 2013; Figure 2). Importantly, the cellular circadian rhythms of *Cry1/Cry2*-deficient SCN were synchronized during the neonatal period but became desynchronized around postnatal day 21, where circadian behavioral rhythms could be measured. Furthermore, synchronized circadian rhythms in neonatal *Cry1/Cry2*-deficient SCN were desynchronized without VIP signaling. *Avp* expression was also attenuated in *Cry1/Cry2*-deficient SCN in both neonatal and adult SCN (Ono et al., 2016; Figure 2B). These results indicate that VIP and AVP are responsible for CRY-independent and -dependent cellular coupling, respectively, and that these neuropeptides are differentially involved in the maturation of circadian networks in the SCN.

## Discussion

Since the first report demonstrating the importance of the SCN for circadian rhythms in behavior (Moore and Eichler, 1972; Stephan and Zucker, 1972), SCN circadian rhythms have been recorded in slice culture as well as *in situ*. Since culturing neonatal SCN is easier than culturing adult SCN, researchers have used neonatal SCN to explain phenotypes of behavior of mice lacking specific genes. However, recent studies have demonstrated that neuronal networks in the SCN are modulated during development. CRY1 and CRY2 are involved in the maturation of neuronal networks in the SCN during postnatal development. Although the functions of neuropeptides in the SCN cellular network have been investigated, their roles as output signals regulating peripheral circadian oscillators have not been clearly identified. Recent advances in various methodologies allow us to manipulate cell type- and developmental stage-specific gene expression, cellular signaling, and neuronal activity, which can help identify mechanisms for the developmental aspects of circadian rhythms in the SCN.

## Author Contributions

DO and SH wrote the manuscript with support of KH. All authors contributed to the article and approved the submitted version.

## Conflict of Interest

The authors declare that the research was conducted in the absence of any commercial or financial relationships that could be construed as a potential conflict of interest.
