# Corrigendum: Roles of Neuropeptides, VIP and AVP, in the Mammalian Central Circadian Clock

**DOI:** 10.3389/fnins.2021.810796

**Published:** 2021-12-07

**Authors:** Daisuke Ono, Ken-ichi Honma, Sato Honma

**Affiliations:** ^1^Department of Neuroscience II, Research Institute of Environmental Medicine, Nagoya University, Nagoya, Japan; ^2^Department of Neural Regulation, Nagoya University Graduate School of Medicine, Nagoya, Japan; ^3^Research and Education Center for Brain Science, Hokkaido University Graduate School of Medicine, Sapporo, Japan

**Keywords:** circadian rhythm, suprachiasmatic nucleus, AVP, VIP, neuronal coupling, synchronization, entrainment

In the original article, the reference for Maywood et al. (2011) was incorrectly written as “Maywood, E. S., Chesham, J. E., Meng, Q. J., Nolan, P. M., Loudon, A. S. I., and Hastings, M. H. (2011). Tuning the period of the mammalian circadian clock: additive and independent effects of CK1 epsilon(Tau) and Fbxl3(Afh) mutations on mouse circadian behavior and molecular pacemaking. J. Neurosci. 31, 1539–1544.” It should be “Maywood, E. S., Chesham, J. E., O'Brien, J. A., and Hastings, M. H. (2011). A diversity of paracrine signals sustains molecular circadian cycling in suprachiasmatic nucleus circuits. *Proc. Natl. Acad. Sci. U. S. A*. 108, 14306–14311.”

The authors apologize for this error and state that this does not change the scientific conclusions of the article in any way. The original article has been updated.

## Publisher's Note

All claims expressed in this article are solely those of the authors and do not necessarily represent those of their affiliated organizations, or those of the publisher, the editors and the reviewers. Any product that may be evaluated in this article, or claim that may be made by its manufacturer, is not guaranteed or endorsed by the publisher.

